# Tolerability and Pharmacokinetic Evaluation of Inhaled Dry Powder Tobramycin in Children with Cystic Fibrosis

**DOI:** 10.3390/pharmaceutics17030347

**Published:** 2025-03-07

**Authors:** Anne M. Akkerman-Nijland, Paul Hagedoorn, Bart L. Rottier, Floris Grasmeijer, Henderik (Erik) W. Frijlink, Mathijs van Luin, E. ter Weijden, Peter J. Merkus, Daan J. Touw, Onno W. Akkerman, Gerard H. Koppelman

**Affiliations:** 1 Department of Pediatric Pulmonology and Pediatric Allergology, Beatrix Children’s Hospital, University Medical Center Groningen, University of Groningen, 9713 Groningen, The Netherlands; annemakkerman@gmail.com (A.M.A.-N.); b.l.rottier@umcg.nl (B.L.R.); g.koppelman@umcg.nl (G.H.K.); 2 Groningen Research Institute for Asthma and COPD (GRIAC), University Medical Center Groningen, University of Groningen, 9713 Groningen, The Netherlands; 3 Department of Pharmaceutical Technology and Biopharmacy, University of Groningen, 9713 Groningen, The Netherlands; p.hagedoorn@rug.nl (P.H.); f.grasmeijer@rug.nl (F.G.); h.w.frijlink@rug.nl (H.W.F.); 4 PureIMS B.V., 9301 Roden, The Netherlands; 5 Department of Clinical Pharmacy, University Medical Center Utrecht, 3584 Utrecht, The Netherlands; m.vanluin-2@umcutrecht.nl (M.v.L.); e.terweijden@umcutrecht.nl (E.t.W.); 6 Amalia Children’s Hospital, Radboud University Medical Center, 6525 Nijmegen, The Netherlands; peter.merkus@radboudumc.nl; 7 Department of Clinical Pharmacy & Pharmacology, University Medical Center Groningen, University of Groningen, 9713 Groningen, The Netherlands; d.j.touw@umcg.nl; 8 Department of Pulmonary Diseases and Tuberculosis, University Medical Center Groningen, University of Groningen, 9713 Groningen, The Netherlands

**Keywords:** dry powder, tobramycin, cystic fibrosis

## Abstract

**Background:** *Pseudomonas aeruginosa* (*Pa*) is the predominant pulmonary pathogen in persons with Cystic Fibrosis (CF). Nebulization with tobramycin or colistin is mostly applied but has a significant treatment burden. Dry powder (DP) inhalation may offer an attractive alternative. The aim of this study was to assess local tolerability and the systemic pharmacokinetic parameters of increasing doses of dry powder tobramycin. **Methods**: This was a local tolerability and pharmacokinetic evaluation pilot study DP tobramycin of three different doses inhaled through the Cyclops (30, 60, 120 mg) in ten persons with CF, aged 6–18 years, compared to nebulization of tobramycin solution. **Results**: Both nebulization of tobramycin in solution and inhalation of dry powder tobramycin were well tolerated. None of the participants showed a significant drop in FEV_1_ after inhalation. The only two adverse events were cough and bad taste in, respectively, 20% and 13% of all inhalations, compared to 10% cough and 60% bad taste with nebulization. Systemic tobramycin levels were not detected after 30 mg, detected only in 10% after 60 mg and in 30% after 120 mg, compared to 80% after nebulization. **Conclusions**: Inhalation of dry powder tobramycin using the Cyclops is well tolerated, with no significant drop in FEV_1_, and only mild adverse events of cough and bad taste. We found only a few detectable systemic tobramycin levels after inhalation of dry powder tobramycin. We recommend that future studies should focus on the relation between dose and inhaler resistance in different pediatric age groups.

## 1. Introduction

Cystic Fibrosis (CF) is a chronic progressive disorder and the most common life-shortening autosomal recessive disease among Caucasian populations [1]. *Pseudomonas aeruginosa* (*Pa*) is associated with an increased number of exacerbations and accelerated loss of lung function, leading to increased morbidity and mortality [2,3,4,5]. The prevalence of *Pa* increases with age: around 35% of persons with CF (PwCF) have a chronic *Pa* infection by their early twenties [6,7]. Preventing, limiting and treating (chronic) infection with *Pa* is therefore crucial in the management of CF, because this is likely to improve survival and quality of life.

Since the introduction of CFTR modulator therapies, the bacterial burden of typical CF pathogens may change in time. However, ivacaftor therapy in PwCF and G551D-CFTR mutations resulted in a reduction in sputum *Pa*, but none of the subjects eradicated their *Pa* and, after the first year, *Pa* densities rebounded [8]. Moreover, for individuals with rarer CF mutations, CFTR modulator therapies are not available to date and some patients may not tolerate or respond to CFTR modulator therapy [9]. Thus, monitoring of microbiology and optimization of antimicrobial therapy remains important in the treatment of CF.

Current treatment guidelines for PwCF with a *Pa* infection recommend antibiotic inhalation therapy with tobramycin, colistin, levofloxacin or aztreonam, regularly combined with oral macrolides [10,11]. All of these inhaled antibiotics are equally effective for treatment of chronic *Pa* infection in PwCF [12], with a recent Cochrane review concluding that the best evidence available is for tobramycin [13]. Inhaled tobramycin has been shown to be effective in eradication of early *Pa* infection in children with CF. Inhaled tobramycin improves lung function, reduces hospitalization rates and improves quality of life in patients with CF with chronic *Pa* infection [14,15,16,17,18]. Pulmonary administered antibiotics have the advantage of facilitating high drug concentrations at the target site in the lung, while minimizing systemic exposure and toxicity [19].

The most frequently investigated and applied method of pulmonary administration of antibiotics is nebulization [19,20,21,22,23]. Although effective, this nebulization technique for inhalation of antibiotics in solution is a complex and time-consuming burden to patients [19,23]. Administration time per dose is approximately 10–15 min, with additional time for cleaning and sterilization [19,24]. In addition, nebulizers require space and an external power source, which can be problematic during traveling. This may negatively influence adherence, thereby limiting treatment efficacy in real life settings [25]. Furthermore, nebulization brings the risk of auto-re-infection of the patient (contamination of nebulization fluid and/or device) [26,27,28]. Inhalation of antibiotics as dry powder can be an interesting alternative, as it has several advantages over nebulization, such as shorter administration time, higher stability than solutions needed for nebulization, in general, and the lack of requirement of a power source, making it simple and more easy to carry on [29,30].

A relatively new dry powder inhaler is the Cyclops, which is a high-dose disposable dry powder inhaler (DPI) and its combination with colistin has currently an orphan drug status according to the EMA and FDA [31,32,33]. Dry powder tobramycin inhalation via the Cyclops was well tolerated in adults with non-CF bronchiectasis [33]. Moreover, the predecessor of the Cyclops, the Twincer, was preferred above nebulization among a group of adult PwCF with an average adherence of 92.5% [29]. DPIs can also be used in children with CF from the age of 5 years and older [34]. Dosages in adults are probably not applicable to children, since there are age-dependent variations in the anatomy of the airways [35,36], which may affect pulmonary deposition. Also, the inhalation pattern differs between adults and children. The peak inspiratory flow rate and the inhalation volume of children is lower than in adults, and inhalation time is shorter [36,37].

Therefore, the aim of this study was to assess local tolerability and the systemic pharmacokinetic parameters of increasing doses of dry powder tobramycin administered using the Cyclops in children between 6 and 18 years in comparison to nebulization of tobramycin solution.

## 2. Materials and Methods

### 2.1. Study Design and Participants

This study was a multicenter, single ascending study with ten PwCF, aged between 6 and 18. The study was initiated at the CF Center of the University Medical Center Groningen (UMCG). Study participants were recruited via the CF outpatient clinics of the UMCG and the Radboud University Medical Center in Nijmegen. Participants nebulized tobramycin solution in a dose of 300 mg and tobramycin dry powder per inhalation using the Cyclops in single doses starting with 30 mg and ascending to 60 mg and 120 mg with a wash-out period between doses of at least 7 days. In and exclusion criteria are listed in Table 1. Participants already on treatment with tobramycin inhalations had their study visits in the month when they were off tobramycin with at least 72 h after the last tobramycin inhalation. The study protocol was approved by the Dutch Central Committee on Research involving Human Subjects (CCMO) under protocol number NL60250.000.17. Children of 12 years and older and all parents provided written informed consent. The study was registered at www.clinicaltrials.gov (NCT03485456).

### 2.2. Study Drugs

For nebulization, tobramycin solution for inhalation (Tobramycin Steri-Neb, 300 mg/5 mL, Brocacef BV, Maarssen, The Netherlands) was used at a dose of 300 mg, which is the licensed dose in children with CF. The nebulized tobramycin was administered via the PARI LC Plus nebulizer with a PortaNeb compressor. For the dry powder formulation of tobramycin, tobramycin base was obtained from Spruyt Hillen BV (IJsselstein, The Netherlands) and spray dried at the Martini Hospital Pharmacy (Groningen, The Netherlands), following previously described procedures [33]. Each prototype Cyclops contained a blister with a nominal dose of 30 mg tobramycin; the 60 and 120 mg doses were administered as, respectively, 2 and 4 successive inhalations with 30 mg of tobramycin. The doses were chosen based on a theoretical deposition efficiency of the Cyclops (in vitro study results) and the number of doses (1-2-4) that could be inhaled without becoming too much of a burden for the child. The formulation and inhaler are extensively described elsewhere, including its dispersion performance [33]. In summary, the dose compartment of Cyclops was hand-filled with 30 mg of pure spray dried tobramycin base to which 20 mg of a coarse lactose sieve fraction was added to enhance dose emission. The prototype Cyclops has a resistance to airflow of 0.06 kPa^0.5^·min·Ln^−1^, which corresponds to an inhalation flow rate of around 36 L/min at a pressure drop of 4 kPa. At this flow rate, the inhalable fine particle fraction < 5 μm of the emitted aerosol measured by laser diffraction technique is around 85–90% [33].

### 2.3. Inhalation Instruction

The study participants received both instructions on how to nebulize and how to handle the Cyclops for an optimal inhalation maneuver [33,38]. Nebulization was continued until the nebulizer began to sputter. When a participant started to cough during nebulization, the machine was turned off briefly and restarted after the coughing stopped. Instruction and training were conducted using an empty prototype Cyclops connected to a laptop, with in-house developed software application (labVIEW, National Instruments, Groningen, The Netherlands) for recording and processing of flow curves generated through the device. A Cyclops containing tobramycin was handed to the participant, only when consistent flow curves meeting the criteria for optimal inhaler performance was obtained. Inspiratory flow parameters were recorded during each drug administration.

### 2.4. Local Tolerability

The primary objective was to assess local tolerability of dry powder tobramycin inhaled by the Cyclops, compared to nebulization of tobramycin. Local tolerability was assessed by spirometry, combined with active questioning and passive monitoring of adverse events. The Microlab MK8 spirometer (CareFusion, Hoechberg, Germany) was used. Spirometry was performed before inhalation (T0) and 15 (T1), 30 (T2) and 90 (T3) minutes after inhalation. A drop in FEV_1_ of 10% or more compared to baseline FEV_1_ was considered clinically relevant. Active questioning for adverse events was performed directly after tobramycin inhalation and every time a blood sample was drawn. Possible adverse events asked for were cough, bad taste, hoarseness, expectoration, throat pain, dyspnea, nausea, tinnitus or other symptoms.

### 2.5. Systemic Exposure

The secondary objectives were to assess the systemic exposure of tobramycin and measurement of inspiratory flow parameters. To determine the pharmacokinetic analysis, blood samples were collected after nebulization and inhalation with a finger prick for dried blood spot (DBS). Samples were collected using Whatman 903^®^ filter paper and BD microtainer^®^ automated lancets for finger prick sampling as described earlier [39]. Careful instruction was given to clean the hands before blood was taken to avoid contamination with tobramycin. Samples were taken 1, 2, 4, 6 and 8–12 h after inhalation (between 8 and 12 h depending on the participant’s bedtime). The DBS were dried for approximately 24–48 h in open air and afterwards stored at −25 degrees Celsius until analysis. Tobramycin was measured in the DBS using a validated liquid chromatography-tandem mass spectrometry method, performed in the laboratory of the hospital pharmacy of the University Medical Center Utrecht. The lower limit of quantitation (LLOQ) of tobramycin in the DBS was 0.1 µg/mL. If applicable, the first tobramycin concentration below the LLOQ preceding (absorption phase) or following (elimination phase) a value above the LLOQ was considered to be 50% of this LLOQ for pharmacokinetic calculations. Any earlier time points with results below the LLOQ in the absorption phase or later time points below the LLOQ in the elimination phase were treated as missing values. The area under the concentration time curve from t = 0 to t = 8–12 h (AUC_0–8/12_) was calculated by the log-linear trapezoidal rule using the KinFIT function in the pharmacokinetic software package MW/Pharm (Mediware, Groningen, The Netherlands). The maximum serum concentration (C_max_) was read from the measured tobramycin concentrations.

### 2.6. Delivered Dose

The delivered dose with nebulization was calculated from the loading dose of 300 mg of tobramycin in the nebulizer minus the residue of tobramycin in the nebulizer after nebulization. The delivered dose with inhalation was computed from the weighed dose of the blisters and the inhaler residue determined by chemical analysis. We used a 2,4,6-Trinitrobenzene Sulfonic Acid (TNBSA) assay to chemically quantify the amount of tobramycin retained in the Cyclops DPIs.

### 2.7. Pressure Drop of the Cyclops

A differential pressure gauge (Sitrans P250, Siemens, München, Germany) was used to measure the pressure drops generated across the inhaler, after prior pressure drop versus flow rate calibration with a thermal mass flow meter (Brooks Smart Mass Flow Meter 5863S, Hatfield, PA, USA). Inhaler instrumentation was performed without changing the inhaler resistance or interfering with the aerosol delivery. During drug administration, the inspiratory flow rate was recorded to ascertain that the participants generated a 4 kPa pressure drop—corresponding with the target flow rate of 34 L/min. When it seemed that inhalation would be below the required flow rate, participants were encouraged to inhale more forcefully.

## 3. Results

**Figure 1:**
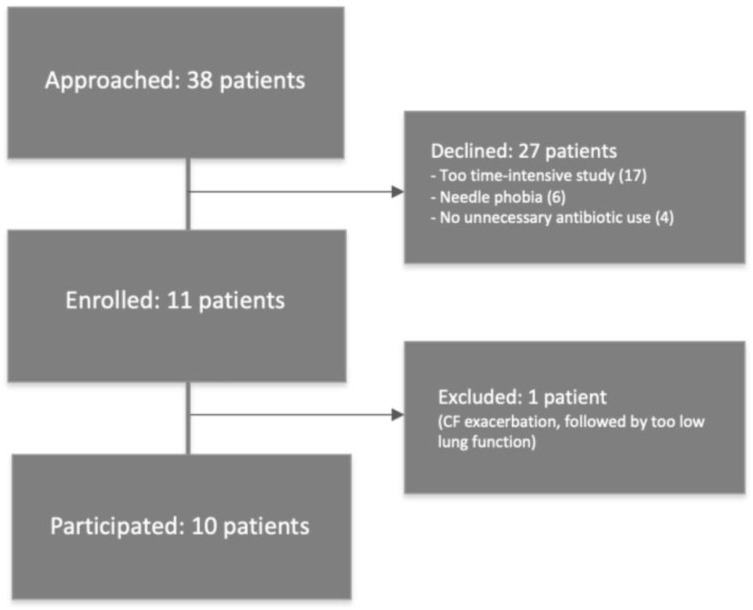
Study flowchart.

Between 2018 and 2020, a total of 38 PwCF were approached to participate in the study, of whom 11 persons consented to participate (Figure 1). One of the enrolled persons was later excluded due to an ongoing CF exacerbation. Reasons for the other 27 PwCF not to participate included ‘too time-intensive study’ (17 times), ‘needle phobia’ (6 times) and ‘no unnecessary antibiotic use’ (4 times).

Baseline clinical characteristics of the ten participants who completed the study are displayed in Table 2. The mean age (range) was 11.5 (7.4–17.6) years old. One of the participants had used inhaled dry powder tobramycin (Podhaler) prior to the start of the study, and eight had used nebulization of tobramycin in solution. Training of performing a correct inhalation maneuver was successful in all participants.

### 3.1. Tolerability

**Figure 2:**
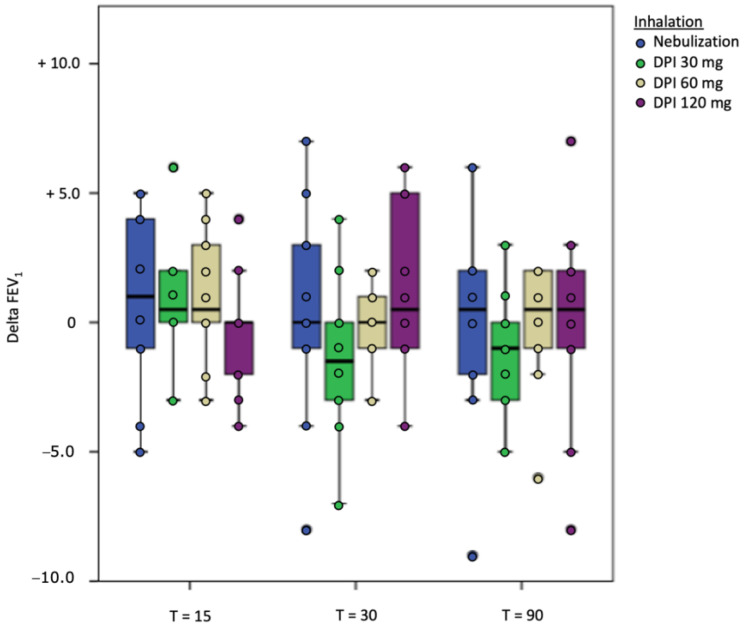
Delta FEV1 per time point on the different study days.

Both nebulization of tobramycin in solution and inhalation of dry powder tobramycin were well tolerated (Table 3). None of the participants showed a significant drop in FEV_1_ of 10% or more compared to baseline FEV_1_, neither for nebulization nor for all doses of the DPI (Figure 2). After nebulization, the maximal drop in FEV_1_ was 9% in one participant after 90 min. The maximal measured FEV_1_ drop after inhalation (120 mg tobramycin dry powder) was 8% in one patient, also 90 min after inhalation.

Bad taste and cough were the only two reported adverse events. Evaluating all three different doses of dry powder tobramycin, three out of ten participants reported cough, one participant reported cough after all three doses of dry powder, one participant complained of cough after the dry powder doses of 60 mg and 120 mg and one participant reported cough only after the first dose of 30 mg but did not mention cough after inhalation of the higher dosages. Thus, cough was reported in 20% of inhalations. All cough events were transient and reported to be mild in intensity. Bad taste after inhalation was reported by two participants four times in total (13%), with one participant having a bad taste after all three dosages and one only after the highest dose (120 mg). After nebulization, one participant reported cough and six of the ten participants (60%) complained of bad taste.

### 3.2. Pharmacokinetic Analysis

**Figure 3:**
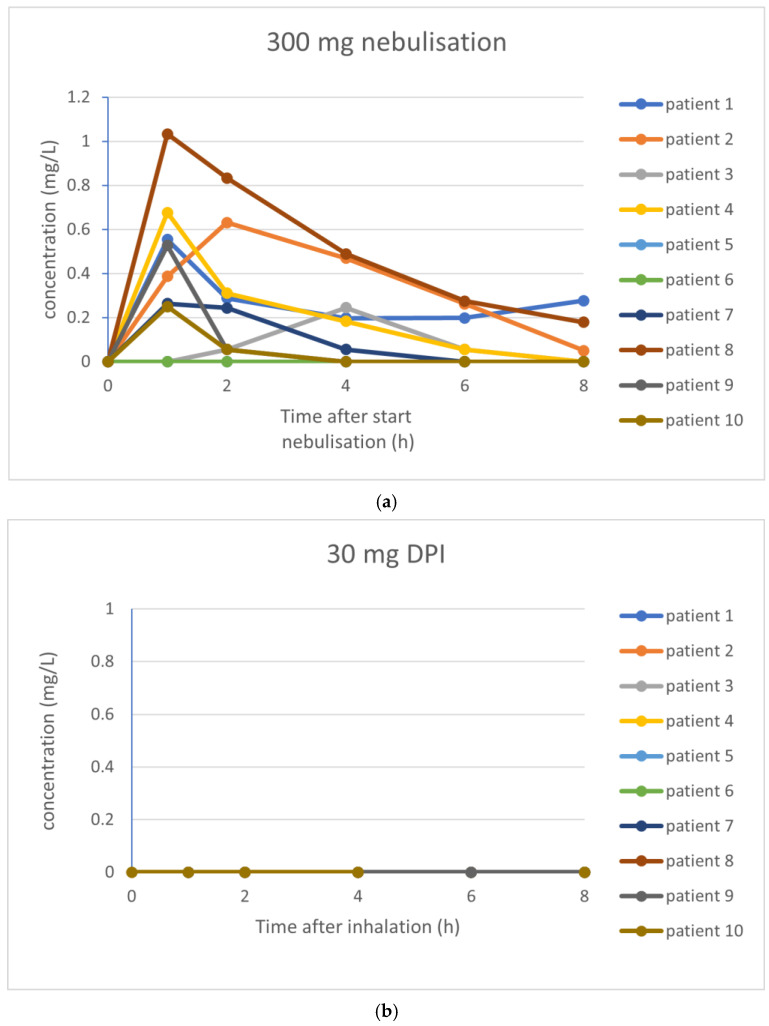

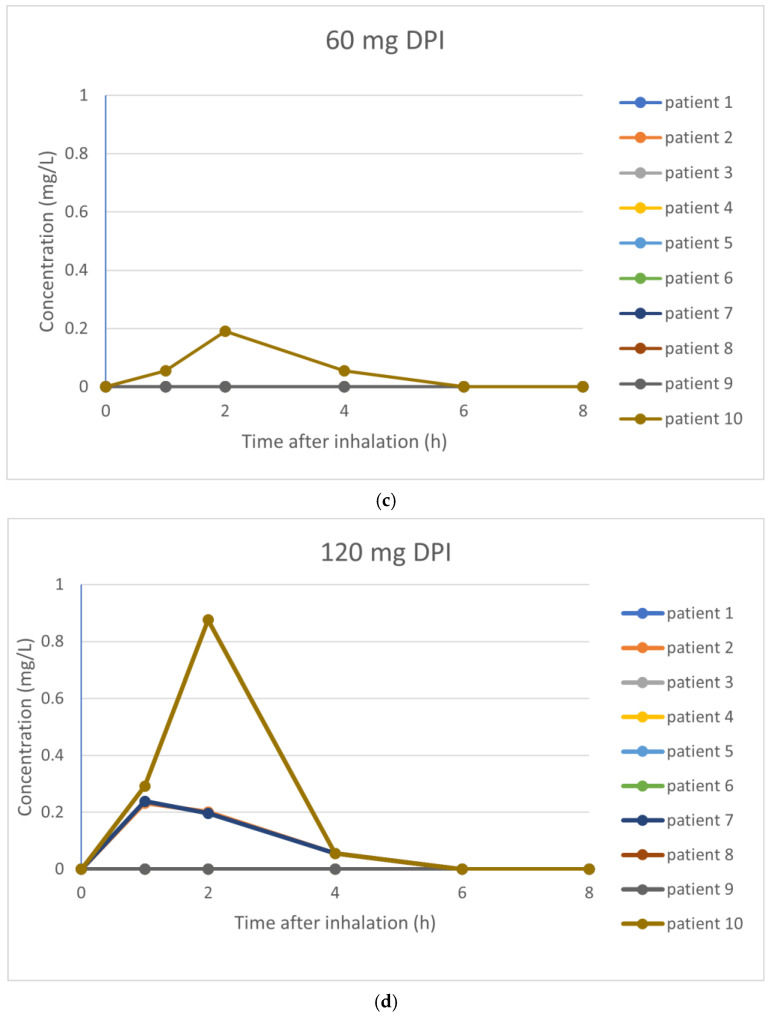
(**a**–**d**) PK profile curves per inhalation. (**a**) PK profile curves per participant with 300 mg nebulization of tobramycin. (**b**) PK profile curves per participant with 30 mg inhalation of dry powder tobramycin. (**c**) PK profile curves per participant with 60 mg inhalation of dry powder tobramycin. (**d**) PK profile curves per participant with 120 mg inhalation of dry powder tobramycin.

The pharmacokinetic parameters investigated are summarized in Table 4. For tobramycin nebulization of 300 mg, an average C_max_ of 0.42 mg/L was found, with an AUC_0–8_ of 1.30 mg·h/L. After inhalation of 30 mg tobramycin, concentrations of tobramycin were below the lower limit of quantitation in all participants. Inhalation of 60 mg tobramycin resulted in one patient with measurable tobramycin levels, whereas inhalation of 120 mg tobramycin resulted in three patients with measurable tobramycin levels. See Figure 3 for the PK profile curves per inhalation.

Large inter-individual differences of the measured delivered dose were observed (see Table 4). For nebulization, the mean delivered fraction of the dose was 40.0% (median delivered dose 134.8 mg; range 63.6–170.9 mg). The mean emitted fraction of the different doses of dry powder tobramycin was 57%.

### 3.3. Recording of Inspiratory Flow

Table 5 shows the characteristics measured through the recording of the inspiratory flow curves. Nine out of ten patients generated a pressure drop of >4 kPa, corresponding with the target flow rate of 34 L/min. Recorded flow curves on the fourth visit (DPI 120 mg) gave a mean pressure drop of all patients of 4.73 kPa. Only one patient did not achieve the targeted pressure drop, with a mean pressure drop over all inhalations of 3.3 kPa.

## 4. Discussion

The aim of this study was to investigate the local tolerability and pharmacokinetic evaluation of increasing doses of dry powder tobramycin using the prototype Cyclops in children with CF and compare these to nebulized tobramycin in solution. Inhalation of dry powder tobramycin was well tolerated. None of the participants showed a significant drop in FEV_1_ ≥ 10% and the prevalence of adverse events using dry powder inhalation of tobramycin was low. Systemic tobramycin levels were not detected in participants after 30 mg; they were detected in only 10% of the participants after 60 mg and in 30% after 120 mg, compared to 80% after nebulization.

The fact that none of the participants showed a drop of FEV_1_ > 10% compared to baseline FEV_1_ after inhalation is remarkable. Geller reported an incidence of 11% of patients with a drop in FEV_1_ ≥ 10% after inhalation with TOBI^®^ Podhaler^®^ [40]. In the EAGER trial, a drop in FEV_1_ ≥ 20% compared to baseline was found in 5.2% of patients in the Podhaler group [24]. Also, after nebulization, no significant FEV_1_ drop was found in our study, whereas other studies showed incidences of 5.3% and 10% [24,40]. After inhalation of dry powder tobramycin, the percentage of cough was 20% in this study, which is similar to the percentage of cough found in the study of Geller (20%, 13/66). In the EAGER trial, 48.4% of the participants reported cough after inhalation of dry powder tobramycin [24]. The experience of bad taste among participants in our study was lower after inhalation (13%). In the study of Geller, 17% of the participants complained of bad taste after dry powder inhalation [40].

In our study, systemic tobramycin levels were detected in only 1 participant out of 10 after inhalation of 60 mg dry powder tobramycin and in 3 participants after 120 mg, compared to in 8 participants after nebulization. Another study applying dry powder inhalation of tobramycin (both in children and adults; 7–50 years of age) showed nearly identical C_max_ and AUC for 300 mg tobramycin nebulization compared to 4 × 28 mg tobramycin dry powder inhalation [40]. They even found measurable tobramycin concentrations at their lowest dose of 2 × 14 mg of dry powder tobramycin (with an LLOQ of serum tobramycin of 0.05 mg/L, instead of the 0.1 mg/L we used). In addition, in an adult population with non-CF bronchiectasis, tobramycin powder inhaled via the Cyclops showed serum tobramycin concentrations that were above the limit of detection with doses of 30 mg (mean serum tobramycin concentration of 0.105 mg/L) [33]. However, also in that study, the C_max_ values of dry powder tobramycin were lower compared to studies with the Podhaler in CF patients and healthy volunteers.

The limited systemic availability of tobramycin after inhalation in this study is difficult to explain due to lack of data on deposition of the inhaled tobramycin. For higher emitted doses, an optimal inspiratory flow rate, the inhaled volume and the inspiration time are important [31]. Furthermore, extrathoracic deposition tends to be higher in children compared to adults [41]. Deposition in the oropharynx probably does not lead to contributing absorption into the systemic circulation, as tobramycin will be swallowed and hardly any absorption takes place from the gastrointestinal tract into the blood [42,43]. A study investigating the requirements for a pediatric dry powder inhaler showed high incidence of obstructions in the oral cavity during inhalation (up to 90%) observed with a sinuscope [44]. In our study, we observed a relatively large retention of tobramycin after inhalation. Also, the computed inhaled volumes in this study are relatively low, ranging from 0.42 to 1.71 L (measured with DPI 120 mg), which was on average 33% of the FVC. However, the one patient who did not reach the targeted pressure drop of >4 kPa, with also a rather short inspiration time (1.8 s) and low inhaled volume (0.49 L) (all leading to possible poor lung deposition) had the highest measurable C_max_ and AUC of all patients after inhalation of 120 mg dry powder tobramycin. Another possible reason for the pharmacokinetic findings could lie in the storage and analysis of the DBS. However, a stability analysis to assess the degradation of tobramycin on DBS at room temperature was performed. This showed a degradation of 11% at a mean storage of 36 h at room temperature and thus is not expected to contribute to significant differences. Furthermore, the storage of the DBS in the freezer was not expected to yield any negative effect, since stored tobramycin in DBS is very stable in the freezer [45].

Our study is one of the very few pharmacokinetic evaluation studies of dry powder inhalation in children and has strengths and limitations. The number of participants was limited to only ten, but previous studies with this number of subjects demonstrated that this sample size is adequate to address our research question [33,38,40,46,47]. Next to that, the age range for participants from 6 to 18 years is very broad. Due to differences in anatomy, it is probably better to choose for a smaller range, though this study still provides valuable information. Moreover, we did not collect data on extrathoracic and intrathoracic deposition, which would be of high interest to include in future studies.

Finally, our study also raised an important issue: how to investigate drug concentrations at the site of infection in the lung. We may have to reconsider the necessity to measure the systemic pharmacokinetic parameters for inhaled antibiotics or even use it for bioequivalence as systemic exposure is not predictive of clinical efficacy. Furthermore, sputum concentrations, which have been used frequently in the past, are highly variable and therefore not sufficiently reliable for pharmacokinetic investigations [48]. Thus, studies with clinical endpoints rather than pharmacokinetic proxy measures should be considered, yet costly to perform in an academic setting.

## 5. Conclusions

This study demonstrates that inhalation of dry powder tobramycin using the prototype Cyclops is well tolerated, with no significant drop in FEV_1_, and only mild adverse events of cough and bad taste. We observed only a few detectable systemic tobramycin levels after inhalation of dry powder tobramycin. We recommend that future studies should focus on the relation between dose and inhaler resistance in different pediatric age groups.

## Figures and Tables

**Table 1:** Inclusion and exclusion criteria.

Inclusion criteria	- Clinical diagnosis of CF and a positive sweat test or two CF-causing mutations - Age 6–18 years - Ability to breathe through a mouthpiece and use the Cyclops - Ability to perform pulmonary function tests reliably and reproducibly - Written informed consent (child and/or parents)
Exclusion criteria	- Acute exacerbation of pulmonary infection - FEV_1_ < 60% - Subjects with known or suspected renal, auditory, vestibular of neuromuscular dysfunction, or with severe, active hemoptysis - History of adverse events on previous tobramycin or other aminoglycoside use - No concurrent use of cisplatin, cyclosporine, amphotericin B, cephalosporins, polymyxins, vancomycin and NSAID’s

**Table 2:** Patient characteristics.

**Sex, *n* (%)**	
Male	6 (60.0)
Female	4 (40.0)
**Age in years, mean (range)**	11.5 (7.4–17.6)
**BMI, mean (range)**	18.0 (13.8–24.3)
**CFTR mutation, *n* (%)**	
Homozygote_Phe508del	6 (60.0)
Heterozygote_Phe508del	4 (40.0)
Other	-
**Comorbidities, *n* (%)**	
Cystic fibrosis-related diabetes (CFRD)	2 (20.0)
Cystic fibrosis-related liver disease (CFLD)	3 (30.0)
Pancreas insufficiency	7 (70.0)
Osteoporosis	1 (10.0)
**Forced Expiratory Volume in one second**	
Percentage of predicted, mean (range, ±SD)	94.0 (78.0–121.0, ±15.3)
Absolute (litres), mean (range, ±SD)	2.1 (1.3–3.0, ±0.6)
**Coinfection with pathogens, *n* (%)**	
*Pseudomonas aeruginosa*	
*Chronic*	1 (10.0)
*Intermittent*	0
*Free*	3 (30.0)
*Never*	6 (60.0)
*Staphylococcus Aureus*	7 (70.0)
*Haemophilus Influenza*	1 (10.0)
*Streptococcus Pneumoniae*	0
*Aspergillus*	2 (20.0)
*Acinetobacter*	1 (10.0)
*Stenotrophomonas Maltophilia*	1 (10.0)
*Burkhholderia*	0
*Non-tuberculosis Mycobacteria*	0

**Table 3:** Local tolerability.

	Nebulization 300 mg	DPI 30 mg	DPI 60 mg	DPI 120 mg
Maximal drop in FEV_1_ in %pred	9%	7%	6%	8%
Median drop in FEV_1_ in %pred (range)	0 (0 to −9)	−2 (0 to −7)	−1.5 (0 to −6)	−0.5 (0 to −8)
Mean delta FEV_1_ in %pred(range)	0.5 (−9 to +7)	−1.8 (−7 to +6)	−0.9 (−6 to +5)	0.2 (−8 to +7)
Cough	1/10	2/10	2/10	2/10
Bad taste	6/10	1/10	1/10	2/10

**Table 4:** Delivered dose and pharmacokinetic parameters.

	Nebulization 300 mg	DPI 30 mg	DPI 60 mg	DPI 120 mg
Mean delivered dose, in mg	134.8	16.1	30.1	79.5
Delivered dose min–max, in mg	63.6–170.9	11.1–25.8	12.8–49.1	54.0–83.9
Delivered dose 25–75th percentile	77.8–151.9	13.5–18.3	21.3–35.2	57.4–83.9
AUC_0–8_ (h mg/L)	1.30 ± 1.34	Not detectable	0.45 (1/10 patients)	1.00 ± 0.62 (3/10 patients)
C_max_ (mg/L), ±SD	0.42 ± 0.33	Not detectable	0.19 (1/10 patients)	0.45 ± 0.37 (3/10 patients)

**Table 5:** Recording of inspiratory flow curves.

	Inhalation Dry Powder Tobramycin 120 mg
Inhaled total volume, in liters, median (min–max)	0.65 (0.49–1.64)
Pressure drop, in kPa, mean (SD)	4.73 (±0.71)
Total inspiration time, in seconds, median (min–max)	1.79 (±1.48–4.28)

## Data Availability

Data is contained within the article.

## Abbreviations

The following abbreviations are used in this manuscript:

AUC	Area under the curve
CCMO	Central Committee on Research involving Human Subjects
CF	Cystic Fibrosis
Cmax	Maximum concentration
DBS	Dried blood spot
DPI	Dry powder inhalation
FEV1	Forced expiratory volume in 1 s
LLOQ	Lower limit of quantitation
PwCF	Patients with Cystic Fibrosis
UMCG	University Medical Center Groningen

## References

[1] David P.B. (2001). Cystic fibrosis. Pediatr. Rev..

[2] Aebi C., Bracher R., Liechti-Gallati S., Tschäppeler H., Rüdeberg A., Kraemer R. (1995). The age at onset of chronic Pseudomonas aeruginosa colonization in cystic fibrosis: Prognostic significance. Eur. J. Pediatr..

[3] Ballmann M., Rabsch P., von der Hardt H. (1998). Long-term follow up of changes in FEV1 and treatment intensity during Pseudomonas aeruginosa colonisation in patients with cystic fibrosis. Thorax.

[4] Emerson J., Rosenfeld M., McNamara S., Ramsey B., Gibson R.L. (2002). Pseudomonas aeruginosa and other predictors of mortality and morbidity in young children with cystic fibrosis. Pediatr. Pulmonol..

[5] Schaedel C., de Monestrol I., Hjelte L., Johannesson M., Kornfält R., Lindblad A., Strandvik B., Wahlgren L., Holmberg L. (2002). Predictors of deterioration of lung function in cystic fibrosis. Pediatr. Pulmonol..

[6] Govan J.R.W., Deretic V. (1996). Microbial pathogenesis in cystic fibrosis: Mucoid Pseudomonas aeruginosa and Burkholderia cepacia. Microbiol. Rev..

[7] FitzSimmons S.C. (1993). The changing epidemiology of cystic fibrosis. J. Pedatr..

[8] Hisert K.B., Heltshe S.L., Pope C., Jorth P., Wu X., Edwards R.M., Radey M., Accurso F.J., Wolter D.J., Cooke G. (2017). Restoring cystic fibrosis transmembrane conductance regulator function reduced airway bacteria and inflammation in people with cystic fibrosis and chronic lung infections. Am. J. Respir. Crit. Care Med..

[9] Saiman L. (2019). Improving outcomes of infections in cystic fibrosis in the era of CFTR modulator therapy. Pediatr. Pulmonol..

[10] Mogayzel P.J., Naureckas E.T., Robinson K.A., Brady C., Guill M., Lahiri T., Lubsch L., Matsui J., Oermann C.M., Ratjen F. (2014). Cystic Fibrosis Foundation pulmonary guideline. Pharmacologic approaches to prevention and eradication of initial pseudomonas aeruginosa infection. Ann. Am. Thorac. Soc..

[11] Langton Hewer S.C., Smyth A.R. (2017). Antibiotic strategies for eradication Pseudomonas aeruginosa in people with cystic fibrosis. Cochrane Rev..

[12] Elborn J.S., Vataire A.L., Fukushima A., Aballea A., Khemiri A., Moore C., Medic G., Hemels M.E.H. (2016). Comparison of Inhaled Antibiotics for the Treatment of Chronic Pseudomonas aeruginosa Lung Infection in Patients With Cystic Fibrosis: Systematic Literature Review and Network Meta-analysis. Clin. Ther..

[13] Smith A., Rowbotham N.J. (2022). Inhaled anti-pseudomonal antibiotics for long-term therapy in cystic fibrosis. Cochrane Database Syst. Rev..

[14] Ramsey B.W., Pepe M.S., Quan J.M., Otto K.L., Montgomery A.B., Williams-Warren J., Vasiljev-K M., Borowitz D., Bowman C.M., Marshall B.C. (1999). Intermittent administration of inhaled tobramycin in patients with cystic fibrosis. Cystic Fibrosis Inhaled Tobramycin Study Group. N. Engl. J. Med..

[15] Murphy T.D., Anbar R.D., Lester L.A., Nasr S.Z., Nickerson B., VanDevanter D.R., Colin A.A. (2004). Treatment with tobramycin solution for inhalation reduces hospitalizations in young CF subjects with mild lung disease. Pediatr. Pulmonol..

[16] Sawicki G.S., Signorovithc J.E., Zhang J., Latremouille-Viau D., von Wartburg M., Wu E.Q., Shi L. (2012). Reduced mortality in cystic fibrosis patients treated with tobramycin inhalation solution. Pediatr. Pulmonol..

[17] Bowman C.M. (2002). The long-term use of inhaled tobramycin in patients with cystic fibrosis. J. Cyst. Fibros..

[18] Moss R.B. (2002). Long term benefits of inhaled tobramycin in adolescent patients with cystic fibrosis. Chest.

[19] Yahya H. (2025). Dallal Bashi, Rachel Mairs, Rand Murtadha and Vicky Kett. Pulmonary Delivery of Antibiotics to the Lungs: Current State and Future Prospects. Pharmaceutics.

[20] Gibson R.L., Emerson J., McNamara S., Burns J.L., Rosenfeld M., Ynker A., Hamblett N., Accurso F., Dovey M., Hiatt P. (2003). Significant microbiological effect of inhaled tobramycin in young children with cystic fibrosis. Am. J. Respir. Crit. Care Med..

[21] Wiesemann H.G., Steinkamp G., Ratjen F., Bauernfeind A., Przyklenk B., Döring G., von der Hardt H. (1998). Placebo-controlled, double-blind, randomized study of aerosolized tobramycin for early treatment of Pseudomonas aeruginosa colonization in cystic fibrosis. Pediatr. Pulmonol..

[22] Proesmans M., Vermeulen F., Boulanger L., Verhaegen J., De Boeck K. (2013). Comparison of two treatment regimens for eradication of Pseudomonas aeruginosa infection in children with cystic fibrosis. J. Cyst. Fibros..

[23] Tiddens H.A.W.M., Bos A.C., Mouton J.W., Devadason S., Janssens H.M. (2014). Inhaled antibiotics: Dry or wet?. Eur. Respir. J..

[24] Konstan M.W., Flume P.A., Kappler M., Chiron R., Higgins M., Brockhaus F., Zhang J., Angyalosi G., He E., Geller D.E. (2011). Safety, efficacy and convenience of tobramycin inhalation powder in cystic fibrosis patients: The EAGER trial. J. Cyst. Fibros..

[25] Sawicki G.S., Sellers D.E., Robinson W.M. (2009). High treatment burden in adults with cystic fibrosis: Challenges to disease self-management. J. Cyst. Fibros..

[26] Riquena B., Monte L.F.V., Lopes A.J., Ferreira da Silva-Filho L.V.R., Damaceno N., da Silva Aquino E., Marostica P.J.C., Ribeiro J.D. (2019). Microbiological contamination of nebulizers used by cystic fibrosis patients: An underestimated problem. J. Bras. Pneumol..

[27] Peckham D., Williams K., Wynne S., Denton M., Pollard K., Barton R. (2016). Fungal contamination of nebuliser devices used by people with cystic fibrosis. J. Cyst. Fibros..

[28] Bell J., Alexander L., Carson J., Crossan A., McCaughan J., Mills H., O'Neill D., Moore J.E., Millar B.C. (2020). Nebuliser hygiene in cystic fibrosis: Evidence-based recommendations. Breathe.

[29] Akkerman-Nijland A.M., Grasmeijer F., Kerstjens H.A.M., Frijlink H.W., van der Vaart H., Vonk J.M., Hagedoorn P., Rottier B.L., Koppelman G.H., Akkerman O.W. (2020). Colistin dry powder inhalation with the Twincer™: An effective and more patient friendly alternative to nebulization. PLoS ONE.

[30] Akkerman-Nijland A.M., Yousofi M., Rottier B.L., van der Vaart H., Burgerhof J.G.M., Frijlink H.W., Touw D.J., Koppelman G.H., Akkerman O.W. (2020). Eradication of Pseudomonas aeruginosa in cystic fibrosis patients with inhalation of dry powder tobramycin. Ther. Adv. Respir. Dis..

[31] Hoppentocht M., Akkerman O.W., Hagedoorn P., Frijlink H.W., De Boer A.H. (2015). The Cyclops for pulmonary delivery of aminoglycosides; a new member of the Twincer family. Eur. J. Pharm. Biopharm..

[32] Community Register of Orphan Medicinal Products. https://ec.europa.eu/health/documents/community-register/html/o2997.htm.

[33] Hoppentocht M., Akkerman O.W., Hagedoorn P., Alffenaar J.W., van der Werf T., Kerstjens H., Frijlink H., de Boer A. (2016). Evaluation of inhaled dry powder tobramycin free base in non-cystic fibrosis bronchiectasis patients. PLoS ONE.

[34] Lexmond A.J., Hagedoorn P., Frijlink H.W., Rottier B.L., de Boer A.H. (2017). Prerequisites for a dry powder inhaler for children with cystic fibrosis. PLoS ONE.

[35] Golshahi L., Finlay W.H. (2012). An idealized child throat that mimics average pediatric oropharyngeal deposition. Aerosol Sci. Technol..

[36] Amirav I., Newhouse M.T. (2012). Deposition of small particles in the developing lung. Paediatr. Respir. Rev..

[37] Lindert S., Below A., Breitkreutz J. (2014). Performance of dry powder inhalers with single dosed capsules in preschool children and adults using improved upper airway models. Pharmaceutics.

[38] de Reus Y.A., Hagedoorn P., Sturkenboom M.G.G., Grasmeijer F., Bolhuis M.S., Sibum I., Kerstjens H.A.M., Frijlink H.W., Akkerman O.W. (2022). Tolerability and pharmacokinetic evaluation of inhaled dry powder hydroxychloroquine in healthy volunteers. PLoS ONE.

[39] Martial L.C., Hoogtanders K.E.J., Schreuder M.F., Cornelissen E.A., van der Heijden J., Joore M.A., Van Maarseveen E.M., Burger D.M., Croes S., Brüggemann R.J.M. (2017). Dried blood spot sampling for tacrolimus and mycophenolic acid in children: Analytical and clinical validation. Ther. Drug Monit..

[40] Geller D.E., Konstan M.W., Smith J., Noonberg S.B., Conrad C. (2007). Novel tobramycin inhalation powder in cystic fibrosis subjects: Pharmacokinetics and safety. Pediatr. Pulmonol..

[41] Carrigy N.B., Ruzycki C.A., Golshahi L., Finlay W.H. (2014). Pediatric in vitro and insilico models of deposition via oral and nasal inhalation. J. Aerosol Med. Pulm. Drug Deliv..

[42] Westerman E.M., De Boer A.H., Le Brun P.P., Touw D.J., Frijlink H.W., Heijerman H.G.M. (2007). Dry powder inhalation of colistin sulphomethate in healthy volunteers: A pilot study. Int. J. Pharm..

[43] Whatley W.S., Chandra R.K., MacDonald C.B. (2006). Systemic absorption of gentamicin nasal irrigatons. Am. J. Rhinol..

[44] Lexmond A.J., Kruizinga T.J., Hagedoorn P., Rottier B.L., Frijlink H.W., De Boer A.H. (2014). Effect of inhaler design variables on paediatric use of dry powder inhalers. PLoS ONE.

[45] Summary of Product Characteristics, Emit® 2000 Tobramycin Assay. https://imgcdn.mckesson.com/CumulusWeb/Click_and_learn/SDS_SEDISE_REAGENT_EMIT_2000_TOBRAMYCIN_30ML.pdf.

[46] Le Brun P.P., De Boer A.H., Mannes G.P., de Fraîture D.M.I., Brimicombe R.W., Touw D.J., Vinks A.A., Frijlink H.W., Heijerman H.G.M. (2002). Dry powder inhalation of antibiotics in cystic fibrosis therapy: Part 2. Inhalation of a novel colistin dry powder formulation: A feasibility study in healthy volunteers and patients. Eur. J. Pharm. Biopharm..

[47] Pilcer G., Goole J., Van Gansbeke B., Blocklet D., Knoop C., Vanderbist F., Amighi K. (2008). Pharmacoscintigraphic and pharmacokinetic evaluation of tobramycin DPI formulations in cystic fibrosis patients. Eur. J. Pharm. Biopharm..

[48] Stockmann C., Roberts J.K., Yellepeddi V.K., Sherwin C.M.T. (2015). Clinical pharmacokinetics of inhaled antimicrobials. Clin. Pharmacokinet..

